# Dental Society

**Published:** 1860-10

**Authors:** 


*Dental Society.*—A new society was formed under the name of the Kentucky State Dental Association, W. D. Stone, President. Some few subjects were discussed; members appointed to read essays, and adjourned to reassemble in Louisville the second Tuesday of April, 1861. B.

